# Editorial: Multiscale modeling for the liver

**DOI:** 10.3389/fbioe.2023.1179980

**Published:** 2023-04-13

**Authors:** Harvey Ho, Vahid Rezania, Lars Ole Schwen

**Affiliations:** ^1^ Auckland Bioengineering Institute, The University of Auckland, Auckland, New Zealand; ^2^ Physical Sciences Department, MacEwan University, Edmonton, AB, Canada; ^3^ Fraunhofer Institute for Digital Medicine MEVIS, Bremen, Germany

**Keywords:** liver, modeling, multiscale, transplantation, paracetamol (acetaminophen), hepatotoxicity, microstructure

## Introduction

**FIGURE 1:**
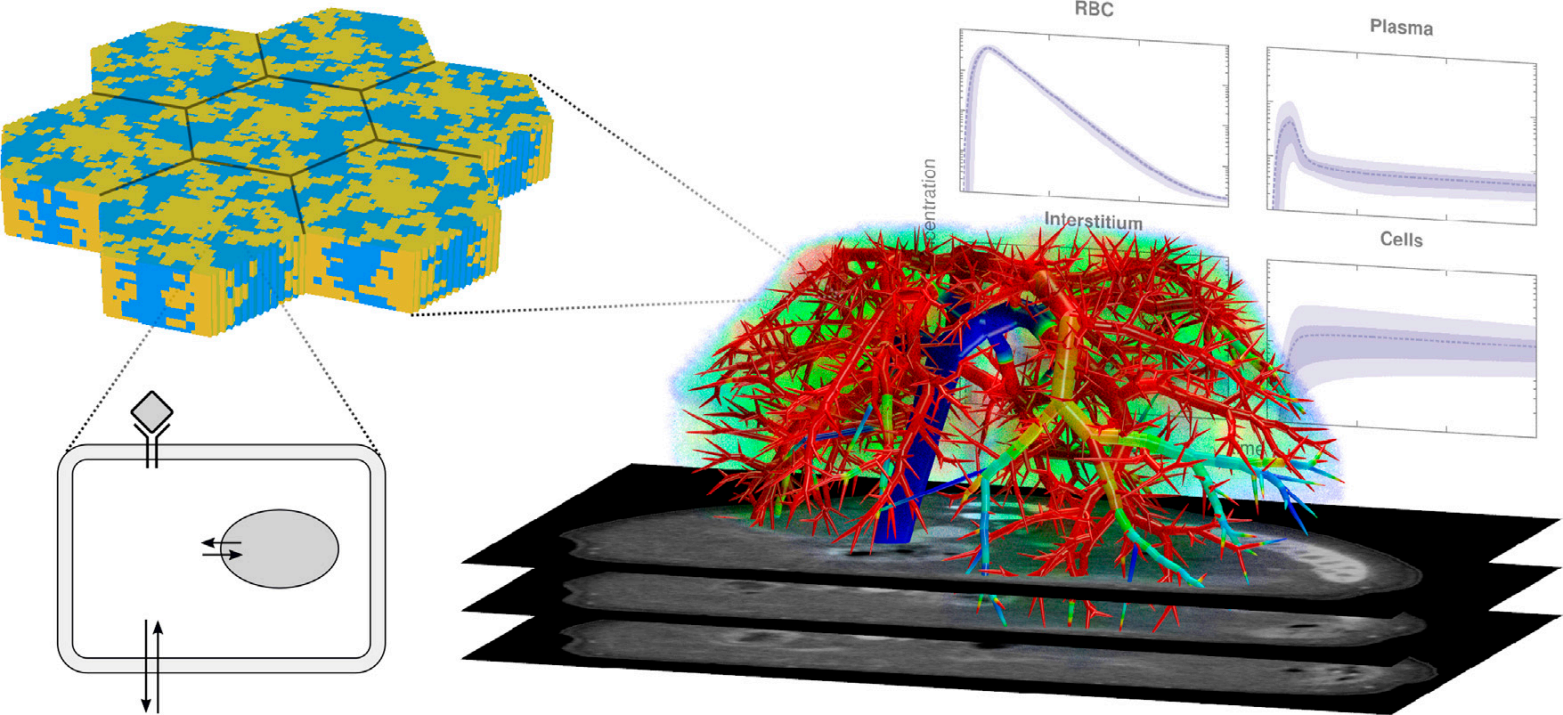
An investigation of hepatic functions requires an understanding of multiple spatial and temporal scales. Perfusion connects the individual cells to the organism, transporting nutrients, xenobiotics, and metabolites. Functions of cells may depend on their locations, the uptake and excretion of compounds across hepatocyte membranes may need the assistance of transporter proteins. CT slices, vascular tree, plots in the background: adapted from https://doi.org/10.1371/journal.pcbi.1003499.g001 (license CC-BY 4.0).

The liver is the central metabolic organ in the mammalian body. Hepatic functions need to be understood from a multiscale perspective, i.e., at the organism, organ, lobular, cellular, and molecular levels that can span vastly different timeframes, e.g., from milliseconds of molecular events to months or years of chronic disease formation (cf. Figure 1). Computational models coupled with experimental measurements and clinical observations provide a viable and cost-effective means to investigate hepatic function. The Research Topic collected some recent results, clinical observations, and updated reviews or perspectives; the seven contributions are briefed below.

### Pharmacokinetics of acetaminophen and induced hepatotoxicity

Hepatotoxicity induced by acetaminophen is one of the major causes of acute liver failure. Dichamp et al. used two mechanistic models, i.e., a well-mixed compartment pharmacodynamic model and a detailed liver lobule model to investigate the *in vitro* to *in vivo* extrapolation (IVIVE) of toxicity. While the detailed model could incorporate heterogeneous mechanisms and data (e.g., enzyme distribution), the authors found the well-mixed model sufficient to predict IVIVE.

Maeda et al. implemented a zonated model of glucose, cysteine, and acetaminophen metabolism in healthy infants, healthy adults, and obese adults. Simulations employ a hybrid of dynamic simulation and metabolic flux analysis. Including the cross-talk between metabolic pathways and the respective zonation of metabolic properties, the model allows an *in silico* investigation of both effects of acetaminophen-induced liver damage on glucose metabolism and the impact of nutritional status on drug-induced damage.

### Hemodynamics in infants receiving liver transplantation

Biliary atresia is a congenital liver disease and remains the most common indication for liver transplantation in children. Chen et al. investigated the peri-operative hemodynamics changes in 41 infants receiving living donor liver transplantation. The authors measured the flow velocities and resistance index in hepatic arteries and portal veins via ultrasonography, and found that the largest hemodynamic change occurred on the first day after transplantation. Hemodynamics stabilized gradually after the 14th-day post-operation. The hepatic arterial buffer response, i.e., the hepatic arterial flow compensates for the changes in portal flow, was obvious on the first-day post-operation.

### Hybrid particle-flow CFD modeling

Bomberna et al. investigated a novel computational methodology to reduce the run time of computational fluid dynamics (CFD) simulations for hemodynamics and microsphere delivery during liver radioembolization. The authors used truncated hepatic arteries, improved the derivation of arterial perfusion-based boundary conditions for CFD simulations, and employed patient-specific composite particle release grids. This way, computational time was reduced by up to 37% without losing accuracy in the predicted microsphere distribution.

### Microstructure of the liver

Coombe et al. developed a fully discretized multi-phase continuum lobule model (fluid in sinusoid and tissue), averaged to represent an averaged lobule, and combined with hepatic vascultures via a gridded dual continuum model to describe a whole-liver model. The authors reported that diffusive transfer had a huge effect on paclitaxel distribution. One of the main achievements is that by upscaling the grid size, they were able to reduce the simulation running time while preserving accuracy.

Verma et al. survey imaging techniques capable of capturing the dynamics of liver regeneration to develop and parameterize computational liver regeneration models. Compared to CT, MRI, and ultrasound at the organism scale, obtaining morphological and physiological data at smaller spatial scales is more challenging, in particular in 3D and *in vivo*. The authors describe recent advances in microscopic and molecular imaging as well as the software used to analyze the resulting image data so that it can be used as the basis for computational models of different types.

### Infection kinetics of hepatitis B virus

Means et al. provide a perspective of applying multiscale spatial-temporal models to the study of hepatitis B virus infection. The authors suggest that the uneven distribution of oxygen and nutrients along sinusoids in liver lobules should be considered in tandem with the spatial aspects of immune cell localizations, blood filtering, and transporter specializations in such a multiscale framework. The paper provides a review of some of the latest *in silico* models on this front which provide controls over said variations and permit investigating competing hepatic dynamics.

## Discussion and conclusion

The articles in this Research Topic highlight a few challenges in multiscale liver modeling: The spatial heterogeneity of metabolism in liver lobules requires parameterization unevenly assigned to different zones, and is suitable for developing multiscale models. Reducing the computational workload for a multiscale framework is necessary, for which a “level-of-details” approach can be applied, e.g., a pharmacokinetic model at the organism level of multiple organs coupled with a highly detailed multi-cell model at the lobule level. Owing to the limitation of the experimental setup and data sampling time, sufficiently verified *in silico* models are powerful in revealing mechanisms underlying complex hepatic functions. To facilitate a systems biology approach, model reusability, curation, and depository are central for multiscale modeling frameworks.

